# Effect of boric acid on oxidative stress in rats with fetal alcohol syndrome

**DOI:** 10.3892/etm.2014.2164

**Published:** 2014-12-30

**Authors:** IBRAHIM SOGUT, AYSEGUL OGLAKCI, KAZIM KARTKAYA, KEVSER KUSAT OL, MELIS SAVASAN SOGUT, GUNGOR KANBAK, MINE ERDEN INAL

**Affiliations:** 1 Department of Medical Services and Techniques, Vocational School of Health Services, Istanbul Bilim University, Istanbul 34394, Turkey; 2 Department of Biochemistry, Faculty of Medicine, Eskişehir Osmangazi University, Eskişehir 26480, Turkey; 3 Department of Genetics and Bioengineering, Yeditepe University, Istanbul 34755, Turkey

**Keywords:** boric acid, catalase, fetal alcohol syndrome, glutathione peroxidase, malondialdehyde, superoxide dismutase

## Abstract

To the best of our knowledge, this is the first study concerning the effect of boric acid (BA) administration on fetal alcohol syndrome (FAS). In this study, the aim was to investigate prenatal alcohol-induced oxidative stress on the cerebral cortex of newborn rat pups and assess the protective and beneficial effects of BA supplementation on rats with FAS. Pregnant rats were divided into three groups, namely the control, alcohol and alcohol + boric acid groups. As markers of alcohol-induced oxidative stress in the cerebral cortex of the newborn pups, malondialdehyde (MDA), superoxide dismutase (SOD), catalase (CAT) and glutathione peroxidase (GPx) levels were measured. Although the MDA levels in the alcohol group were significantly increased compared with those in the control group (P<0.05), the MDA level in the alcohol + boric acid group was shown to be significantly decreased compared with that in the alcohol group (P<0.01). The CAT activity of the alcohol + boric acid group was significantly higher than that in the alcohol group (P<0.05). The GPx activity in the alcohol group was decreased compared with that in the control group (P<0.05). These results demonstrate that alcohol is capable of triggering damage to membranes of the cerebral cortex of rat pups and BA could be influential in antioxidant mechanisms against oxidative stress resulting from prenatal alcohol exposure.

## Introduction

Depending on its acute or chronic use and the dose, alcohol has toxic effects on the mother and fetus when consumed during pregnancy (1). Its toxic effects have been a topic of investigation for approximately five decades (2,3). At present, fetal alcohol spectrum disorders (FASD) have been shown to include alcohol-related birth defects (ARBD), alcohol-related neurological disorders (ARND) and fetal alcohol syndrome (FAS) (3–5). FAS causes mental retardation and growth deficits together with facial anomalies in newborns from chronic alcohol-consuming mothers (3).

Children born with FAS have certain difficulties including learning, memory, attention span, communication, hearing and vision (6,7). Extreme attention, rehabilitation, education, medical support and major economic resources are required to integrate these children into society (8,9). In order to overcome this social problem, new strategies are required to prevent the teratogenic effects of alcohol on the fetus.

Oxidative stress has been shown to be associated with numerous diseases, including cardiovascular disease, cancer and neurological and endocrinological disorders, and also FAS (10,11). Oxidative stress is the imbalance between the production and breakdown of reactive oxygen species (ROS) by endogenous antioxidants (12). Oxidative stress results in increasing lipid peroxidation (13,14) and also ROS production. As a result, membrane and enzyme function may be damaged via overproduction of ROS (11). Antioxidants such as superoxide dismutase (SOD), catalase (CAT) and glutathione peroxidase (GPx) detoxify ROS.

Boric acid (BA) is an essential trace element for plants, humans and animals to support metabolic events. BA is monobasic but is not a proton donor. Instead, it acts as a Lewis acid, accepting a hydroxyl ion from water and releasing a proton. It has high affinity for *S*-adenosyl methionine (SAM) and oxidized nicotinamide adenine dinucleotide (NAD^+^). BA forms complexes with hydroxyl group-containing glycolipids, glycoproteins and phosphoinositides. By this, it affects membrane integrity, calcium chelator function and redox metabolism. When boron is taken orally, it enters the blood stream quickly and thoroughly. Its urinary excretion rate is ~100% (15,16). Boron can be used for neutron capture therapy in certain types of cancer (17). It decreases the severity and incidence of inflammatory diseases (18). BA affects calcium, magnesium, potassium, vitamin D, aldehyde dehydrogenase, xanthine oxidase, cytochrome b reductase, insulin, estrogen, testosterone, T3, T4, triglycerides and glucose metabolism (19). Apart from its diverse functions, previous studies have shown that BA also has antioxidant activity (19–21).

In the current study, the possible protective effect of BA supplementation was evaluated in prenatally alcohol-exposed rats at the cerebral cortex.

## Materials and methods

### Experimental procedure

Ten adult male and 30 adult female Sprague-Dawley rats weighing between 200 and 250 g were created for breeding in the present study. For mating, a male rat was randomly picked up and placed into a female’s cage. They were placed in a secluded, temperature- and humidity-controlled room (22±3°C and 60±5% respectively) in which a 12:12 h light-dark cycle was maintained. The females were checked each morning for the presence of a vaginal plug. The presence of a vaginal plug was considered as evidence of fertilization, and the day the plug was observed was regarded as embryonic day (E) 0, the first embryonic day. Only in 15 females was a vaginal plug observed, and these were used to set up three experimental groups.

Five pregnant rats were selected for each of the control, alcohol and alcohol + boric acid groups. The animal model of prenatal alcohol consumption was modified from that of Uzbay and Kayaalp (22). The control group was pair-fed with an isocaloric modified liquid diet containing sucrose as a caloric substitute for alcohol (96 g sucrose and 75 ml cows’ milk replaced 60.75 g or 75 ml ethanol). Modified liquid diet (MLD) was given to the alcohol and alcohol + boric acid groups. The MLD comprised 925 ml low fat cows’ milk (Sütaş, Istanbul, Turkey), from 25 to 75 ml alcohol (96.5% v/v ethanol; Merck Millipore, Darmstadt, Germany) and 17 g sucrose (Merck Millipore). The calorific content of the diet was 1,000.7 kcal/l. The weight of the rats was recorded every day, and the daily alcohol intake was also measured. The MLD was prepared daily and given to the animals while fresh. Extra chow or water were not available during the experimental period.

**Figure 1:**
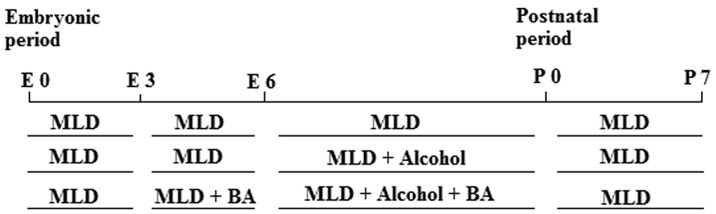
Experimental procedure. E, embryonic day; P, postnatal day; MLD, modified liquid diet; BA, boric acid.

At the beginning of the study, the rats were given MLD without alcohol for 6 days (E0–E5). BA was administered prior to alcohol administration (E3–E5) in order to accustom the rats to drinking the supplements. The dosage of BA (Merck Millipore) was 100 mg/kg (21). Then, a liquid diet with 2.4% alcohol was administered for 3 days (E6–E8). The alcohol concentration was increased to 4.8% during the following 3 days (E9–E11) and finally reached 7.2% over the next 11 days (E11–E22; Fig. 1).

Following delivery, the rats were given MLD without alcohol on postnatal days (P) 0–7. A pool was prepared by selecting two pups from each group of five rats, frozen tissue from this group of pups was used for biochemical analysis. A total of 30 pups (n=10 per group) at P7 were used for the study. Pups were sacrificed by decapitation on day P7. The cerebral cortex was surgically removed. Tissues used for biochemical studies were frozen in liquid nitrogen and kept at −80°C until they were tested. All experiments were carried out in accordance with institutional guidelines for animal welfare (Eskişehir Osmangazi University Animal Care and Use Committee, Eskişehir, Turkey) and were approved by the Ethics Committee of the Medical and Surgical Experimental Research Center of Osmangazi University.

### Blood alcohol concentration (BAC) measurement

On days E15, E18 and E20, every morning 6 and 22 h after the administration of fresh diets, a 20-μl blood sample was taken from the tails of the rats. Plasma was immediately separated following centrifugation and quantified at 340 nm to determine the BAC (NAD-ADH Reagent Multiple Test Vial, Sigma-Aldrich, St. Louis, MO, USA) using a Shimadzu UV-1201 spectrophotometer (Shimadzu Corporation, Kyoto, Japan). For each group, on days E15, E18 and E20, a total of six values were obtained as a result of two applications per day (23). The highest of these values was defined as the peak BAC for each group.

### Biochemical measurement

Lipid peroxidation was quantified at 532 nm by the measurement of malondialdehyde (MDA) reacted with thiobarbituric acid (TBA) according to the method of Ohkawa *et al* (24). The results were expressed in nmol/mg protein. SOD activity was determined according to the method of Winterbourn *et al* (25). One unit of SOD expressed in U/mg protein was designated as the amount of enzyme that inhibits the reduction of nitroblue tetrazolium reduction by 50%. CAT activities were calculated using the method of Beutler (26). The reduction in optical density per minute was determined and the enzyme activity was expressed in U/mg protein. GPx activity in U/g protein was spectrophotometrically determined at 340 nm using the methods of Paglia and Valentine (27). The protein concentration of homogenates gathered from brain tissues were determined using the Bradford assay (28).

### Statistical analysis

SPSS software, version 15.0 for Windows (SPSS, Inc., Chicago, IL, USA) was used for the statistical analysis of biochemical data. In order to assess differences between groups, one-way analysis of variance (ANOVA) and Tukey’s multiple comparison test were used. Results are presented as mean ± standard deviation and P<0.05 was considered to indicate a statistically significant result.

## Results

### MDA levels

**Figure 2:**
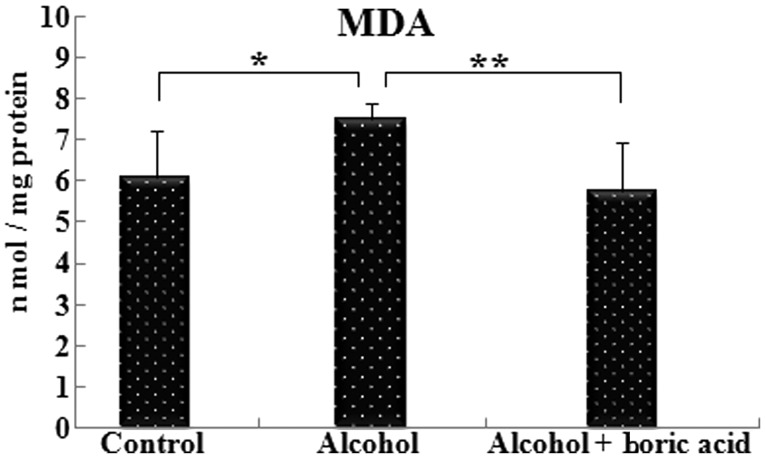
Effect of boric acid on malondialdehyde (MDA) levels in rats exposed to alcohol. ^*^P<0.05; ^**^P<0.01. Data shown are mean ± standard deviation.

MDA levels in the alcohol group (7.53±1.41 nmol/mg protein) were significantly increased (P<0.05) compared with the control level (6.11±1.11 nmol/mg protein). The MDA level in the alcohol + boric acid group (5.74±1.22) exhibited a significant reduction (P<0.01) compared with that in the alcohol group (Fig. 2).

### Enzyme activities

**Figure 3:**
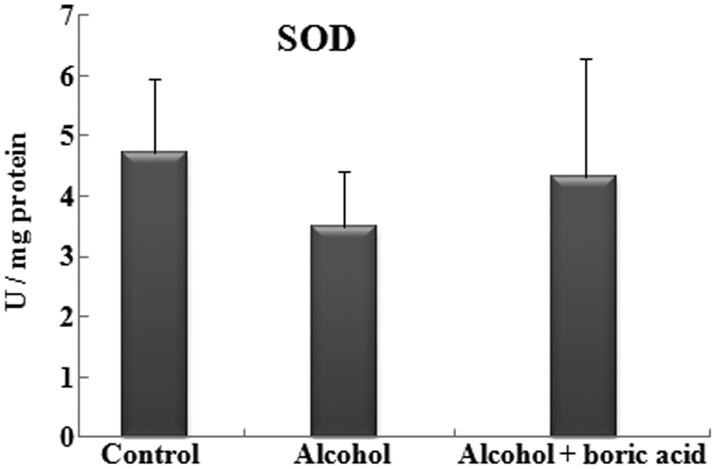
Comparison of superoxide dismutase (SOD) activity among the groups. There were statistically insignificant (P>0.05) differences among the groups. All data are shown as the mean ± standard deviation.

**Figure 4:**
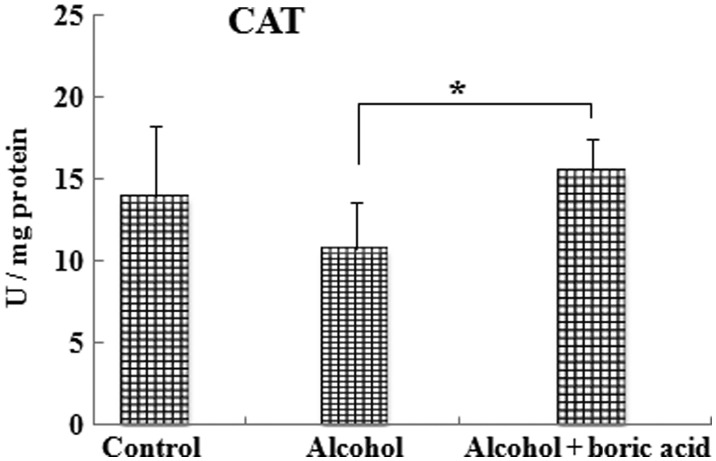
Effect of boric acid on the activity of catalase (CAT) in rats exposed to alcohol. ^*^P<0.05. All data are shown as the mean ± standard deviation.

**Figure 5:**
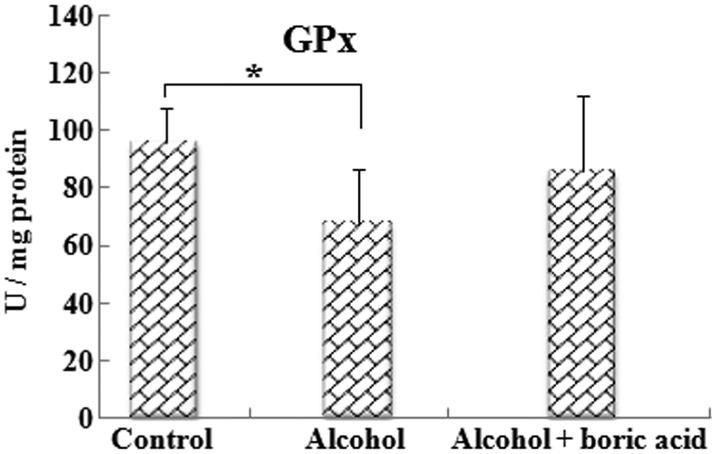
Comparison of glutathione peroxidase (GPx) among the groups. ^*^P<0.05. All data are shown as the mean ± standard deviation.

No statistically significant differences in SOD activities were identified among the groups. The SOD values in the control, alcohol and alcohol + boric acid groups were 4.73±1.22, 3.50±0.92 and 4.31±1.96 U/mg protein, respectively (Fig. 3). The CAT activity of the alcohol + boric acid group (15.55±3.65 U/mg protein) was found to be higher (P<0.05) compared with that of the alcohol group (10.80±2.79 U/mg protein). There was no statistically significant difference (P>0.05) in CAT activity between the control group (13.90±4.34 U/mg protein) and the alcohol group (10.80±2.79 U/mg protein; Fig. 4). GPx activity in the alcohol group (67.49±18.88 U/mg protein) was decreased (P<0.05) as compared with that in the control group (95.70±25.85 U/mg protein). The GPx activity of the alcohol + boric acid group (85.67±29.71 U/mg protein) was not significantly different (P>0.05) from that on the other two groups (Fig. 5).

### Blood alcohol levels

The highest blood alcohol levels recorded in the two alcohol-administered groups were between 175 and 182 mg/dl.

## Discussion

To the best of our knowledge, this is the first study concerning the effect of BA administration on rats with FAS and the possible antioxidant mechanisms. Alcohol may have direct and indirect effects on the brain. The direct effect of alcohol results in diffusion from the lipid barrier of the cell membrane and increased flowability in the membrane. Oxidative and non-oxidative metabolisms are associated with the indirect effects of alcohol (29). Acetaldehyde is generated, and as a result of forming protein adducts, acetaldehyde disrupts mitochondria and restorative enzymes, leading to the formation of reactive oxygen species. Furthermore, acetaldehyde causes a reduction in the number of glutathione groups and hydrophobic regions at the cell membrane and mitochondrial membranes (30). Fatty acid ethyl esters are produced as a result of non-oxidative metabolism, preventing the esterification of cholesterol on neuronal membranes and also disrupting mitochondrial and myelin metabolism (31). It is well-documented that oxidative stress-induced by alcohol-associated mechanisms gives rise to cell damage.

The constitutive formation of oxidants can be balanced by the production of antioxidants at a similar rate. The imbalance between oxidants and antioxidant species causes oxidative stress, resulting from the peroxidation of lipids. MDA is one of the end products in the lipid peroxidation process (32,33). In the present study, prenatal alcohol intake increased the level of lipid peroxidation. These findings are in line with previous fetotoxicity studies (34,35). Enzymatic (SOD, CAT and GPx) and non-enzymatic (thiol and GSH) antioxidants have important roles in preventing the damage resulting from alcohol-induced ROS (11). When female rats were exposed to alcohol during gestation, GPx activity decreased while SOD and CAT activities were not significantly affected (36). In a study concerning free radical formation in rat brains exposed to acute doses of ethanol, it was demonstrated that in the early postnatal period while ROS levels increased, GPx activity decreased (37). Accordingly, chronic alcohol exposure has been shown to decrease cytosolic and mitochondrial GPx activity by 40 and 30%, respectively, in rat liver (38). The steady activity levels of enzymatic antioxidants, namely SOD and CAT, may be due to levels of those enzymes being higher in an adult rat than in a fetus. Furthermore, the activities of these enzymes have been shown to be lower in the brain than other tissues, for example, in the liver and kidney (11).

At present, the complete antioxidant mechanism of BA is not fully understood (39); however, BA is a well-known component of cell membrane functions and enzymatic reactions (40,41). In a previous study, boron supplementation (100 mg/kg/day) was shown to decrease lipid peroxidation by enhancing antioxidant activity (21). In another study, a 100-mg/kg/day dose of BA was also used and shown not to be toxic for rodents when administered for a short period (≤4 weeks) (42). According to the proposed hypothesis, a possible mechanism for the decline of alcohol-induced lipid peroxidation might be the increasing levels of SAM with BA administration. Being a Lewis acid, BA may form complexes with many biological compounds through its hydroxyl groups. It has high affinity for SAM, which is known as a methyl donor in biochemical processes, a metabolite in the transsulfuration pathway and a precursor in the synthesis of polyamines (43). In a previous study, chronic alcohol consumption decreased SAM levels while increasing *S*-adenosylhomocysteine (SAH) levels and decreasing the methylation capacity indicator ratio, SAM/SAH (44). In another study on boron deprivation in the liver, the level of SAM was reported to decrease while the homocysteine level increased (41). As Nielsen (40) indicated, boron balances transmembrane signaling and ion movements with its structural and/or functional properties on the cell membrane. According to this information, in the current study, it is hypothesized that a decline in lipid peroxidation was associated with an increase in the SAM/SAH ratio, resulting in balancing of the cell membrane functions.

In the present study, the blood alcohol level reached 175–182 mg/dl in the alcohol group. This level might lead to impairment in prenatal development since it has been reported that a BAC >100 mg/dl in pregnant rats can impair embryo-fetal development (45). The alcohol + boric acid group had no significant change in SOD and GPx activities compared with the alcohol group. SOD and GPx inactivate superoxide and hydrogen peroxide, respectively. In an earlier study of BA supplementation in rats, SOD activity was not affected in erythrocytes or in heart and liver tissues (21). Paralleling the results of the present study, SOD activity was found to be unchanged in another study conducted to assess the effect of BA on the redox status of the rat liver (46). However, in those studies, BA increased GPx activity. Turkez *et al* demonstrated that when boron compounds were administered at low doses (15 mg/l), the CAT activity of erythrocytes increased, whereas high doses (500 mg/l) of boron led to decreased CAT activities (20). In a study of carbon tetrachloride-induced toxicity in rats, it was shown that BA administration at various doses (50, 100 and 200 mg/kg) increased the CAT enzyme activity in the liver (19). Placental transfer from the mother to fetus and the presence of CAT in the fetus are responsible for the presence of aldehydes in fetal rat brains (47). In the present study, the increasing CAT activity following BA administration may stem from the ability of BA (a Lewis acid) to accept a hydroxyl ion. BA potentially has two crucial functions in this mechanism. Firstly, it detoxifies hydrogen peroxide derived from alcohol by converting it into peroxyboric acid. Secondly, BA converts the CAT enzyme to its native state and maintains the enzyme.

In summary, alcohol-related oxidative stress of cell membranes can be decreased by BA supplementation. According to the results of this study, BA has no impact on blood alcohol levels, but may be a great candidate for use in the protection and regulation of brain membranes. However, future studies are required to evaluate this recovery effect of BA on FAS.
